# Raman Spectroscopy for Probing Pathological Protein Aggregates: Potential and Perspectives for Advanced Diagnostic Applications

**DOI:** 10.3390/ijms27125550

**Published:** 2026-06-19

**Authors:** Alice Gualerzi, Valentina Mangolini, Luana Forleo, Chiara Cabrini, Silvia Picciolini, Marzia Bedoni

**Affiliations:** 1 IRCCS Fondazione Don Carlo Gnocchi ETS, 20148 Milan, Italy; vmangolini@dongnocchi.it (V.M.); lforleo@dongnocchi.it (L.F.); ccabrini@dongnocchi.it (C.C.); spicciolini@dongnocchi.it (S.P.); mbedoni@dongnocchi.it (M.B.); 2 Department of Pathophysiology and Transplantation, University of Milan, 20122 Milan, Italy

**Keywords:** neurodegenerative diseases, α-synuclein, amyloid-β, diagnosis, biomarkers, Raman spectroscopy, surface-enhanced Raman spectroscopy, protein aggregation

## Abstract

Parkinson’s disease and Alzheimer’s disease are currently classified as a major global health burden, sharing a defining pathological hallmark represented by insoluble protein aggregates of α-synuclein (α-syn) and amyloid-β (Aβ), respectively. A defining characteristic of all amyloids is a highly ordered, unbranched filamentous morphology, where individual β-strands align perpendicularly to the filament axis. Despite recent technological advances, direct observation of protein conformational changes and amyloid formation in biological samples remains a challenge as well as the quantification of pathological aggregates in liquid biopsies. This review critically recapitulates the major advances in the application of Raman spectroscopy (RS) and surface-enhanced Raman spectroscopy (SERS) in the investigation of pathological protein aggregates in neurological disorders, with a focus on α-syn and Aβ. We discuss both in vitro structural characterization and the applications to biological and clinical samples, outlining the main challenges for clinical translation, including the need for standardized protocols. Recent achievements in the use of RS and SERS on liquid biopsies and other clinical samples are paving the way for further implementation of Raman-based approaches for the diagnosis of neurodegenerative disorders.

## 1. Introduction

The differential diagnosis of neurodegenerative diseases represents a current unmet clinical need in neurology in striving for early diagnosis and for monitoring of pharmacological and rehabilitation treatment. The emergence of advanced technologies has brought considerable promise to the diagnostic field, but multiple challenges still need to be addressed. Globally, the two most prevalent neurodegenerative disorders are Parkinson’s disease (PD) and Alzheimer’s disease (AD), which currently represent a global health burden [1]. Both conditions are complex multifactorial disorders, but at the molecular level, they are defined by the accumulation of misfolded proteins which can form aggregates and deposits inducing neuronal death and neuroinflammation, leading to the clinical manifestations. In PD, the 140-residue intrinsically disordered (ID) protein α-synuclein (α-syn) undergoes conformational changes, from monomers to toxic oligomers to fibrils that can accumulate in the Lewy bodies [2]. In AD, the 36–43-residue amyloid-β (Aβ) peptide, primarily Aβ40 and Aβ42, aggregates. Aβ can form oligomers, protofibrils, and plaques that tangle together with hyper-phosphorylated tau inducing neuronal cell death [3]. 

The identification of pathological protein aggregates is critical for both PD and AD, but still their detection and quantification are far from trivial in vivo for several reasons. On the one hand, the inaccessibility of the brain requires the quantification of biomarkers in peripheral fluids and tissues, where the low concentrations and the multi-organ expression of the target molecules limit the reliability of the measures and raise the risk of misleading results. On the other hand, from a biochemical point of view, protein aggregates are usually unstable and expose variable epitopes, whereas the production of antibodies generally targets linear epitopes. This results in antibodies prone to nonspecific binding between monomers and oligomers, which challenge both their diagnostic application by conventional immune-based assays and their use as therapeutics [4,5]. For the basic identification of amyloids, several approaches are employed, including microscopy techniques (transmission electron and atomic force microscopy) staining procedures (e.g., Congo red and thioflavin T), nuclear magnetic resonance (NMR) spectroscopy), although most of them remain far from clinical application.

In this context, vibrational spectroscopies are powerful analytical techniques that probe the intrinsic molecular vibrations of a sample, providing detailed information on its chemical composition and structure [6]. Among these, Raman spectroscopy (RS) is one of the most widely used, enabling label-free, non-destructive, and rapid analysis of complex biological samples [7]. By generating characteristic molecular fingerprints, RS allows the simultaneous detection of multiple biomolecules, including proteins, lipids, nucleic acids, and metabolites, without interference from water. This capacity makes RS particularly suitable for the comprehensive analysis of biofluids [8], offering insights into disease-related biochemical alterations while minimizing sample preparation and preserving native molecular information.

Despite its promise, spontaneous RS suffers from inherent weak signal intensity, as only about one in 10^6^–10^8^ photons undergoes Raman scattering. This limitation has led to the development of signal-enhancement strategies, most notably surface-enhanced Raman spectroscopy (SERS), which exploits metallic nanostructures to amplify Raman signals by several orders of magnitude [9]. The principle underlying SERS is that when molecules are adsorbed onto or located near metallic nanostructures, typically composed of gold or silver, the local electromagnetic field generated by the excitation of surface plasmons drastically increases the Raman scattering efficiency. This enhancement can reach factors of 10^6^ to 10^10^, allowing the detection of molecules at very low concentrations.

In this review, we describe major advances in the application of RS and SERS to the probing of pathological protein conformation associated with neurodegenerative disorders. After a first introduction to the principle of conformational studies, the most relevant results in the study of α-syn and Aβ are reported. In conclusion, the limitations and the future perspective for clinical translation are presented.

## 2. Principles of Raman Spectroscopy for Protein Conformation

RS is a vibrational spectroscopic technique based on the inelastic scattering of light, first described by C.V. Raman in 1928 [10]. Within a Raman spectrometer, the monochromatic light generated by a laser source is directed towards the sample. When it interacts with the sample, most photons undergo elastic scattering (Rayleigh scattering), in which their energy and wavelength remain unchanged. However, a small fraction is scattered inelastically, resulting in a shift in photon energy [11] that reflects the vibrational states of the molecules. This phenomenon is registered by a detector, and it is known as the Raman effect, which provides molecular information that enables the identification of chemical bonds. The resulting Raman spectrum is typically represented as a plot of intensity versus Raman shift (expressed in cm^−1^). Each peak corresponds to a specific vibrational mode, providing characteristic molecular information. Importantly, RS is label-free and non-destructive, enabling real-time characterization of samples without the need for additional reagents or extensive preparation.

In the biomedical context, RS has gained considerable attention due to its ability to provide detailed biochemical information from complex matrices such as tissues [12], cells [13,14], and biofluids [15,16,17]. Unlike conventional biochemical assays, Raman spectra capture molecular signatures that reflect proteins, lipids, nucleic acids, and metabolites simultaneously. Indeed, the so-called fingerprint region (600–1800 cm^−1^) contains most of the characteristic peaks associated with proteins, nucleic acids, lipids, and carbohydrates, thereby enabling a comprehensive assessment of cellular and extracellular components [18].

With RS, several vibrational modes can be used for the analysis of peptide structure. By probing the vibrational modes of proteins, researchers can determine the protein secondary structure and identify the transition from functional monomeric states to misfolded aggregates, which is often characterized by specific shifts in the secondary structure.

Indeed, the core potential of RS in clinical proteomics lies in its ability to perform “conformational fingerprinting” by reflecting polypeptide backbone conformations and protein secondary structure in two most informative regions, the Amide I and Amide III vibrational bands [19,20].

Amide I band (1600–1690 cm^−1^) primarily arises from C=O stretching of the peptide bond. This is the most widely exploited band for secondary structure analysis. The typical Raman shifts (cm^−1^) for α-helix and β-sheet structures are 1652–1655 cm^−1^ and 1674–1672 cm^−1^, respectively [16].

Amide III band (1230–1300 cm^−1^) arises from coupled C–N stretching and N–H bending. It has been found to be particularly sensitive to fibril polymorphism and subtle tertiary structural differences. The typical cm^−1^ values for α-helix and β-sheet structures are 1272–1264 cm^−1^ and 1242–1227 cm^−1^, respectively [18], although the Amide III band analysis is often complicated by overlapping bands of side chain vibrations [21].

Overall, the rest of the fingerprint region contains contributions from amino acid side chains, S–S stretching of disulfide bonds, and C–C backbone vibrations, offering additional structural information on tertiary contacts and side-chain environments. Aromatic amino acid side chains (e.g., phenylalanine) provide additional markers, with a prominent “breathing mode” peak at 1000–1004 cm^−1^ and C=C vibrations at 1600 cm^−1^.

## 3. Raman Spectroscopy of α-Synuclein Aggregation

α-syn aggregation is the defining characteristic of PD and other synucleinopathies. Tracking the conformational state of α-syn is challenging due to its transient and heterogeneous nature. α-syn is an ID protein, which means that, in contrast to well-folded proteins, it is not limited to one or a few predominant conformations, but many different conformations can coexist, allowing rapid interconversion. This enables interactions with diverse binding partners and response to a variety of stimuli [22]. Although a central region of α-syn (residues 61–95; NAC region) was identified as the region responsible for toxicity and aggregation propensity, the ID property provides α-syn with a remarkable specificity in binding to biological membranes with selected chemical (charge content, headgroup chemistry) and physical properties (membrane curvature, packing density) that mediate its physiological function [23,24,25]. For α-syn, the membrane binding domain consists of its N-terminal ~100 residues that mediate the ability of the peptide to sense membrane curvature [22,26,27]. Once the peptide interacts at the membrane interface, local rearrangement occurs and α-helix folding is completed, thus making membrane interaction one of the variables impacting on the α-syn conformational state [27,28].

### 3.1. Structural Features of α-Syn Monomers and Fibrils

In solution, α-syn does not adopt a unique conformation, but spontaneously aggregates to form fibrils in vitro that resemble those isolated from patients [29]. To better understand how these structural features relate to aggregation, it is essential to examine the conformational properties of α-syn in both the monomeric and fibrillar states.

**Figure 1:**
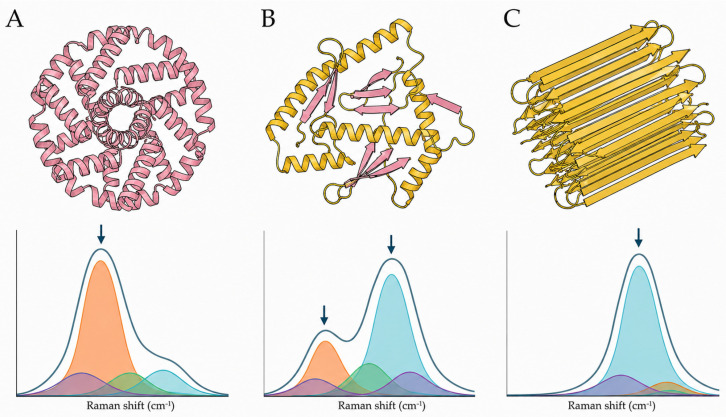
Schematic representation of the conformational and spectral changes of α-syn in aqueous solution. The educational illustration does not report experimental data but schematically represents the structural modifications of the intrinsically disordered protein α-syn that does not adopt a unique, stable conformation but spontaneously aggregates to form fibrils. The changes in conformation, from predominantly α-helix (**A**) to mixed α-helix and β-sheet (**B**) to well-ordered β-sheet (**C**), are reflected by the corresponding variations in the spectral bands of the Amide I region obtained by Raman spectroscopy. The image was obtained with Notebook LM (Gemini 3.5).

α-syn can adopt extensive α-helix and β-sheet conformations, becoming α-helical when bound to micelles or phospholipids and forming β-sheet structure that allows fibril formation at low pH [21]. In solution, the dynamic conformation of α-syn spontaneously aggregates to form fibrils, assembling in well-ordered β-sheets with β-strands running perpendicular to the fibril axis. In this context, RS can serve as a basis for the characterization of conformational changes in secondary structure that are correlated with the changes in the morphology of the aggregates [30] (Figure 1). In aqueous solution at pH 7.5, α-syn was proved to be a mixture of conformations with broad bands at both Amide I and Amide III regions [21]. For the Amide I region, a curve fitting technique was applied to dissect the contribution of α-helical conformation centered at 1654 cm^−1^ band and β-sheet structures at 1667 cm^−1^. The latter conformation was proved to be favored by increasing the methanol concentration that narrows the Amide I band with the peak maximum moving to 1667 cm^−1^ and providing a quantitative, label-free monitor of the monomer-to-fibril transition. The β-conformation induced by methanol also makes the Amide III region sharper, with a new band at 1240 cm^−1^ that substitutes the broader 1252 cm^−1^ band attributed to undefined secondary structure [21].

A landmark study by Flynn demonstrated that RS can characterize structural features of α-syn amyloid fibrils with sensitivity beyond that of conventional approaches [31]. By systematically varying the pH and ionic strength and examining four PD-linked mutations (A30P, E46K, G51D, A53T), they observed distinct spectral differences in the Amide I and Amide III regions. These differences indicated that the secondary structure of α-syn fibrils was strongly modulated by the environmental pH conditions that impact the Amide I band, whereas the mutants exhibited distinctive Amide III features [31]. These reported perturbations in the protein conformation were not apparent in the transmission electron microscopy data, highlighting the superior sensitivity of RS for resolving molecular-level differences between fibril polymorphs.

Having established the ability of RS to resolve α-syn structural features in vitro, its application has been progressively extended to more complex biological systems.

### 3.2. Raman Spectroscopy of α-Syn in Biological Samples

Apart from the structural characterization of standard molecules, few studies have taken advantage of RS to investigate the α-syn conformation and aggregation state in a biologically relevant context.

First, the possibility of combining Raman microspectroscopy with machine learning analysis was applied to cellular models of α-syn aggregation. Using multiple in vitro cell lines (HEK293, Neuro2a, and SH-SY5Y) the effect of treatment with sonicated preformed fibrils (PFF) was tested. RS allowed the tracking of biomolecular changes in the selected in vitro models, induced by the pathological aggregation of α-syn [32]. PFF treatment induced significant increases in the spectral peaks related to β-sheet in all samples. Specifically, increases in peak intensity were seen at 1045 cm^−1^ (β-sheet formation) and 1250 cm^−1^ (Amide III/β-sheet formation), while decreases were seen at 1659 cm^−1^ (Amide I/α-helical structures), suggesting rapid aggregation into β-sheet conformation [32].

To investigate the cellular localization of α-syn fibrils, Raman spectral imaging was applied to human cells (SK-MEL-28) treated with exogenous α-syn fibrils [33]. Watson and colleagues took advantage of isotopic labelling of α-syn amyloid fibrils and the related changes in the peak frequency to distinguish the labeled Amide I band from the Amide I band of endogenous proteins. The study allowed the localization of the fibrils mainly at the cellular periphery, partially colocalizing with endogenous lipids and proteins, and concomitantly obtaining information on the secondary structure (13C=O) [33].

In vivo, RS was also applied to brain and colon tissue sections of a human BAC-SNCA transgenic rat model [34]. RS images of rat tissues were investigated, and the analysis of the Amide I region revealed increased β-sheet content in the transgenic brain tissues compared to the wild-type controls. The prevalence of β-sheet structures together with the changes in the secondary structure of the lipids were interpreted as signs of increased α-syn aggregation and fibrillization process. The promising results obtained using RS to detect signs of fibrillization in complex tissue matrices without labeling led the authors to propose Raman imaging as a new method for peripheral biomarker discovery [34].

Finally, in humans, RS was also used to investigate α-syn in minimally invasive samples. León-Bejarano et al. tested the use of RS for the detection of α-syn aggregates in skin tissue [35]. Using stained paraffin-embedded skin biopsies, the ability to detect changes in the Amide I region in agreement with their theoretical data was demonstrated. Nonetheless the application was tested only on a few samples, suggesting the need for further validation.

## 4. Raman Spectroscopy of Amyloid-β Aggregation

Aβ peptides, specifically Aβ42 and Aβ40, are major components of amyloid deposits in senile plaques and are mainly responsible for the neurodegeneration of AD.

Aβ derives from the proteolysis of the amyloid precursor protein (APP), a ubiquitously expressed glycoprotein that is particularly abundant in the brain. In neurons, APP can act as a trophic factor, being required not only during synaptogenesis, synapse remodeling, and neurite outgrowth but also during neuronal maturation and differentiation [36]. APP metabolism can alternatively undergo the non-amyloidogenic or the amyloidogenic pathway. The former proceeds from the proteolysis of APP on the cell surface by α-secretase and it is followed by γ-secretase, leading to non-toxic peptides. On the contrary, the amyloidogenic sequence produces neurotoxic peptides. It involves cleavage by β-secretase, followed by γ-secretase. The result is the production of Aβ peptides that exhibit peculiar structural properties. Indeed, Aβ is an ID peptide with an amphipathic sequence comprising a polar N-terminal region (residues 1−16) followed by a central hydrophobic core (residues 17−21), a polar region (residues 22−29), and a non-polar C-terminal region (residues 30−40/42). Similar to α-syn, Aβ40 and Aβ42 lack a well-defined structure. Indeed, in physiological conditions, certain regions of Aβ42 are known to exhibit helical propensities and this molecular conformation strongly favors aggregation events, with aromatic residues playing a critical role in the interaction between monomers. They then adopt a β-sheet conformation in the fibrillar forms, as found in amyloid plaques in the brain.

To characterize Aβ structural properties at the molecular level, different strategies have been employed, including NMR studies. An NMR study considered different fragments of Aβ at different pH levels and showed that there is a difference in the conformational preferences of these fragments when in solution as monomers and during amyloidogenesis. NMR showed that the Aβ42 monomer at lower pH can adopt an α-helical conformation, but when the pH is increased, the peptide switches to β-sheet conformation and aggregation occurs [37].

In this framework, RS offers a valuable approach to monitor the conformational alterations in peptides and proteins occurring in the brain of AD, where the accumulation of Aβ42 is only the final outcome of progressive neurodegeneration.

### 4.1. Spectral Fingerprinting of Aβ Aggregates

The typical cross-β architecture of the amyloid fibrils of Aβ generates a characteristic Raman Amide I signature, similar to α-syn [38]. RS cannot determine the molecular conformation of peptides or proteins at the atomic level, but it is highly informative for tracing the global conformational changes occurring during AD progression.

The RS analysis of the Amide I band of Aβ1–42 proved a predominant β-sheet conformation of the peptide. The analysis of the spectrum revealed a Gaussian type band centered at 1667–1670 cm^−1^ (C = O stretching vibration) corresponding to the secondary β-sheet conformation [39,40]. Additionally, the deconvolution of the spectral region between 1600 cm^−1^ and 1700 cm^−1^ showed two minor bands at 1608 cm^−1^ and 1634 cm^−1^. These bands represent less than 10% of the total spectral contribution and can be attributed to the amino acid side chains and the aromatic amino acid ring mode vibration of the peptide [40].

The low abundance of Aβ in peripheral biofluids together with its low intrinsic Raman cross-section in clinically relevant matrices have driven the development of surface-enhanced Raman spectroscopy (SERS) approaches. The SERS effect takes advantage of the molecular proximity of the analyte to plasmonic metal nanostructures, typically gold (Au) or silver (Ag), which amplify the Raman signals by factors of 10^6^–10^10^ [41]. SERS has proven to be highly effective in detecting Aβ even at trace levels in different samples.

In label-free SERS, Ag nanoparticle (AgNP)-decorated carbon fibers have been developed as SERS sensor platforms for the detection of Aβ25-35, which absorbs directly onto the metal nanosubstrates to generate intrinsic fingerprint spectra. By comparing the Raman spectrum of the peptide with the SERS spectrum, a significant increase in the sensitivity of Aβ was achieved (Raman 10 mM vs. SERS 100 μM). As expected, the SERS spectra displayed highly enhanced distinguishable peaks of Aβ with five major vibrational peaks centered at 1600, 1447, 1342, 1216, and 1004 cm^−1^ which correspond to the C–C stretching, CH_2_ bending, CH deformation, C–N stretching, and phenylalanine, respectively. The study highlighted the SERS efficiency towards the trace level detection of Aβ, although no real biological setting was tested [42]. AgNPs were also used to generate SERS hotspots for the detection of Aβ by functionalizing AgNP with a purine-based ligand which was proved to provide non-covalent interactions with the amyloid peptide without hindering its detection by RS [43]. Furthermore, implementing a microfluidic device with purine-AgNP, the SERS spectrum of target Aβ42 was tested as well as the repeatability and reproducibility of the detection. The study allowed the identification of a characteristic peak at 1270 cm^−1^, even though the SERS approach was not tested in real clinical samples.

### 4.2. Raman Spectroscopy of Aβ in Biological Samples

In brain sections from an in vivo mouse model, confocal Raman imaging has been demonstrated as a highly effective, label-free approach for the identification and chemical characterization of Aβ amyloid plaques [44]. The authors showed that the spatial correlation between multiple Raman marker bands enables reliable discrimination of β-sheet-rich amyloid plaques from non-amyloid proteins and the surrounding lipids [44].

**Figure 2:**
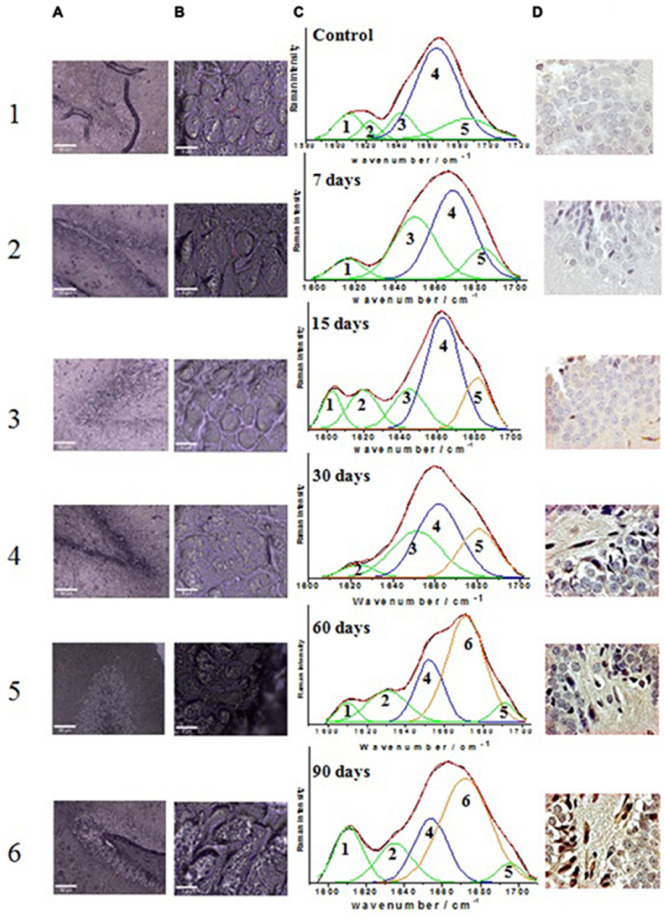
Effect of exposure to low doses of ozone on the relative abundance of different secondary conformations of the Aβ42 peptide [40]. Columns (**A**) and (**B**) show the micrographs of the areas where the punctual Raman spectra were obtained (**A**: 10×; **B**: 100×); column (**C**) shows the tissue Raman spectra in the Amide I spectral interval (1600–1720 cm^−1^); column (**D**) shows the micrographs of immunoreactivity of Aβ42 peptide in the dentate gyrus of hippocampus (40×). Row 1 shows the results of the control group while rows 2, 3, 4, 5, and 6 show the groups exposed to ozone at 7, 15, 30, 60, and 90 days, respectively. Reproduced from Rivas-Arancibia and colleagues, *Front. Mol. Neurosci.*, 2017 [40].

In a model of progressive neurodegeneration, the effect of oxidative stress on amyloid formation was monitored by RS. Following exposure to low doses of ozone, the effects of oxidation over the secondary structure of Aβ42 were observed in deparaffinized sections of rat hippocampus by the deconvolution analysis of the Amide I band [40]. Data showed a predominance of α-helix structures in the hippocampus of control animals, whereas during the neurodegeneration process, the β-sheet secondary structure prevailed. Indeed, the sub-band corresponding to the α-helix secondary structure decreased whereas the 1670–1682 cm^−1^ band assigned to the β-sheet secondary structure prevailed (Figure 2) [40].

RS was also applied to small extracellular vesicles (EVs) obtained from an in vitro AD cell model [39]. EVs are natural nanoparticles released by all body cells that can circulate in all biofluids, mediating intercellular communication [45,46]. The Aβ associated with these vesicles was shown to retain an α-helical conformation, and it was proposed to be the size of a monomer or small oligomer, possibly early-stage oligomers, rather than mature β-sheet fibrils [39]. The concomitant investigation of the lipid structure of EVs also suggested a role of Aβ in altering plasma membrane fluidity, even though the data require further validation.

To detect Aβ42 in human plasma samples, SERS substrates consisting of Au nanowire arrays were fabricated and functionalized to capture and analyze the peptide using the monoclonal 6E10 antibody, which is specific for Aβ42 [47,48]. Although the information regarding the conformational state of the peptide was lost, the coupling of SERS spectra with deep learning models distinguished the oligomerization steps of Aβ42 with outstanding accuracy, suggesting the potential of the SERS based approach to monitor AD progression as well as the effect of Aβ42-targeting treatments. However, the platform presented limitations in achieving extremely high classification accuracy for distinguishing people with AD from healthy controls focusing on Aβ42, while demonstrating better performance when detecting multiple metabolites in plasma [48].

## 5. Applications in Biological and Clinical Samples

The application of RS to clinically relevant biological samples and disease diagnosis has increased rapidly over the past decade as it offers several advantages for clinical translation [49]. The method has the major advantage of providing exhaustive biochemical characterization of the biological sample without staining and labeling procedures, being highly informative, rapid, and sustainable. The Raman spectrum can be used as a highly specific fingerprint representing the diagnostic biomarker itself, accounting for both soluble proteins and insoluble aggregated amyloid fibrils. Thanks to the high sensitivity of the methodology, once the procedure is optimized, RS can theoretically detect even slight biochemical variations or low concentrations of molecules in the selected biofluid. Spectral patterns derived through matrix factorization can identify disease states with high accuracy [15,16,50], even though data are often derived from limited single-centered cohorts. RS is fast, nondestructive, it requires minimal sample amounts (<10 μL of ~15 μM protein fibrils in solution), and it allows for acquisitions in physiological conditions without any impact from water [18,51,52].

Despite the advantages mentioned, in the field of neurodegenerative diseases, the use of RS on clinical samples is still limited (Table 1 and Table 2). Ryzhikova and colleagues applied NIR to blood serum from AD patients, subjects with other neurodegenerative dementias, and healthy controls, achieving over 95% sensitivity and specificity in differentiating the groups [53]. Beyond serum, RS was also applied to blood plasma: Paraskevaidi et al. analyzed plasma from early-stage AD, late-stage AD, dementia with Lewy bodies (DLB), and healthy controls, reaching 84% sensitivity and 86% specificity for early-stage AD versus controls, and notably discriminating AD from DLB with 81% sensitivity and 88% specificity. Thus, they addressed the clinically relevant challenge of differential dementia diagnosis [54]. The same group later extended this approach to cerebrospinal fluid (CSF), where NIR Raman spectroscopy differentiated AD from healthy controls with 84% sensitivity and specificity [55]. Building on the label-free advantage (i.e., the possibility to avoid the use of antibodies), RS was also investigated for the diagnosis of PD using minimally invasive samples. Carlomagno et al. characterized the salivary Raman fingerprint of people with PD, pathological controls and healthy subjects, reaching classification accuracy, sensitivity, and specificity above 97% for single-spectrum attribution [56]. More recently, Garnaik et al. developed a portable Raman spectrometer targeting salivary α-synuclein, reporting a sensitivity of 88.9% and specificity of 83.3%, supporting the feasibility of point-of-care RS devices for PD screening [57]. Within the complex composition of biofluids, EVs have emerged as particularly informative targets, as they carry molecular cargo reflective of their cell of origin. Raman-based profiling of EVs isolated from biofluids were shown to capture disease-specific biochemical signatures, including alterations in proteins, lipids, and nucleic acids. RS was applied to circulating EVs from people with PD provided with reproducible Raman signatures that correlated with disease severity, likely reflecting changes in brain-derived vesicle populations and their molecular cargo [58].

These findings are encouraging and suggest strong performance of the reported methods, yet they remain preliminary, being based on single-center pilot cohorts of limited size that require validation in larger, independent populations.

To improve the performances of the methods, the SERS approach was proposed taking advantage of the simultaneous detection of multiple targets in the biological samples, other than solely amyloid peptides, and demonstrated a better performance of the method when a multiplex approach was proposed. In the context of SERS approaches, it must be mentioned that the pursuit of high-performance analytical methods frequently needs a trade-off between sensitivity and precision. For example, antibody-based approaches may improve detection limits and enable the quantification of analytes at very low concentrations. Nevertheless, the gains in sensitivity can be accompanied by reduced specificity, which is especially relevant for ID proteins. The simultaneous quantification of Aβ42 and phosphorylated tau (p-Tau181) in blood samples, for example, was obtained with SERS coupled with microfluidic chips with a detection limit of 100 fg/mL, representing a clinically actionable format. Zhan and colleagues designed and constructed gold core silica shells to be used in a SERS based lateral flow assay (LFA) [59]. The method was based on antigen–antibody complex formation and on SERS nanotags encoded by different Raman dyes that were used to simultaneously detect multiple AD biomarkers. Although the method was proved to be rapid and ultrasensitive, it still relied on antigen binding affinity and specificity [59].

In the context of neurodegenerative disorders, one of the major obstacles in the development of sensitive and standardized assays to quantify amyloids in biofluids resides in the dependence of most techniques on antigen–antibody recognition. This is particularly true for α-syn detection methods as antibodies generally target linear epitopes of the molecule which are not easily accessible in oligomers due to their transient, heterogeneous, and dynamic nature. This often leads to unwanted cross reaction between monomers and oligomers, making antibody-based assays highly variable and non-reliable [5,25]. A notable advance in the RS detection of Aβ in human clinical samples was described by D’Andrea et al. who proposed the detection of Aβ by SERS after seed amplification assay (SAA) (SAA-SERS) [60]. The SAA exploits the prion-like self-templating property of Aβ oligomers and amplifies limited amounts of Aβ oligomers in biofluids at the expense of synthetic Aβ peptides, which are used as the reaction substrate. In a cohort of people with AD, mild cognitive impairment due to AD (MCI-AD), or other neurological conditions, SAA was performed on cerebrospinal fluid samples and then analyzed using SERS-active substrate based on networks of silver nanowires. The SERS analysis of SAA revealed a high level of intra-sample reproducibility and the combination of SAA-SERS with machine learning analysis efficiently identified pathological Aβ oligomers in the considered groups [60]. This approach enabled the identification of limited amounts of Aβ oligomers in clinical samples suggesting the possibility to obtain early patient stratification and improved diagnostic accuracy; however the limited sample size requires further validation of the method.

**Table 1:** Sensitivity and specificity of Raman spectroscopy-based approaches for the diagnosis of Parkinson’s disease (PD). Studies are grouped by conventional Raman spectroscopy (RS) and surface-enhanced Raman spectroscopy (SERS). AD: Alzheimer’s disease, HC: healthy controls, MVA: multivariate analysis, ML: machine learning models.

	Parkinson’s Disease					
	Sample	Sample Size	Sensitivity	Specificity	Analytical Method	Ref.
**Raman spectroscopy approach**						
Carlomagno et al., 2021	Saliva	23 PD	97%	98%	MVA, ML	[56]
		10 AD				
		33 HC				
**SERS-based approach**						
Garnaik et al., 2025	Saliva	6 PD	89%	83%	ML	[57]
		6 HC				

**Table 2:** Sensitivity and specificity of Raman spectroscopy-based approaches for the diagnosis of Alzheimer’s disease (AD). Studies are grouped by conventional Raman spectroscopy (RS) and surface-enhanced Raman spectroscopy (SERS). N/A: not available, CSF: cerebrospinal fluid. OD: other neurodegenerative diseases, HC: healthy controls, DLB: dementia with Lewy bodies, E: early stage, L: late stage.

	Alzheimer’s Disease					
	Sample	Sample Size	Sensitivity	Specificity	Analytical Method	Ref.
**Raman spectroscopy approach**						
Ryzhikova et al., 2015	Serum	20 AD 18 OD 10 HC	>95%	>95%	MVA, ML	[53]
Paraskevaidi et al., 2018	Plasma	11 AD (E) 15 AD (L) 15 DLB 15 HC	84% (E) 84% (L)	86% (E) 77% (L)	MVA	[54]
Ryzhikova et al., 2021	CSF	21 AD 16 HC	91%	81%	MVA, ML	[55]
Xu et al., 2023 (Systematic Review)	Serum + CSF	Pooled	86%	87%	-	[52]
**SERS-based approach**						
Carlomagno et al., 2020	Serum	10 AD 11 HC	N/A	86%	MVA	[15]
Kim et al., 2024	Plasma	20 AD 20 HC	83%	92%	ML	[48]
D’Andrea et al., 2023	CSF	16 AD 15 OD	88%	77%	ML	[60]

## 6. Translational Challenges and Future Perspectives

**Figure 3:**
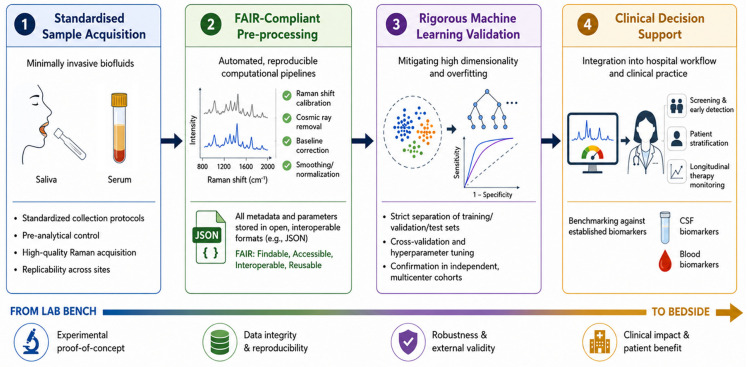
Summary diagram that illustrates the “roadmap” from sample acquisition to clinical decision through standardized pre-processing. The image was obtained with ChatGPT (GPT-5.5).

While Raman-based approaches have already demonstrated strong analytical performance and are progressing toward real-world application, their full translation to routine clinical practice involves a set of well-defined challenges that the field is actively addressing. Most studies applying RS and SERS to neurodegenerative disorders are currently at a proof-of-concept or early validation stage, and acknowledging their current boundaries is essential to guide the next phase of clinical development. These challenges span the standardization of experimental and computational protocols, the rigorous design of classification models, the distinction between conventional RS and SERS, and the comparison with established biomarker platforms, as discussed below (Figure 3).

One of the most significant challenges currently is defining standardized protocols, not only in the experimental phase but also in the computational processing of spectral data. Coca-Lopez et al. [61] highlight that FAIR (Findable, Accessible, Interoperable, Reusable) principles are essential to adopt in the context of RS.

The “Findable” principle requires that acquisition parameters (e.g., laser power, acquisition time), as well as sample and instrumental information, should be properly stored, enriching metadata and utilizing persistent identifiers.

The “Accessible” principle implies the use of standardized communication protocols that are open, free, and universally implementable, ensuring metadata remains accessible even when the spectral data are no longer available.

The “Interoperable” principle underlines the importance of adopting formal and broadly applicable languages and file formats. For example, JSON (JavaScript Object Notation) is a human-readable format which, when paired with code files, aims to separate parameter definition from pipeline implementation.

Finally, the “Reusable” principle requires that data are released with a clear and accessible usage license, as well as with a documented and exact computational workflow.

In this context, it needs to be noted that the way in which preprocessing pipelines are commonly handled often violates the FAIR principles, due to a lack of methodological transparency, standardization, and a high dependence on subjective, user-defined parameters. While literature generally agrees on which preprocessing steps are essential [62,63,64], there is much debate on their order of implementation and whether all of them are necessary (or if extra steps are needed). For instance, while Mostafapour et al. [65] proceeded with interpolation of intensities on the wavenumber axis, followed by spike removal, smoothing, baseline correction, normalization, and both wavenumber and intensity calibration, Bertazioli et al. [66] began with realignment of the Raman shift axis, followed by subtraction of the background signal and noise, cosmic ray removal, and intensity normalization. Guo et al. [67] tried to define a standardized protocol for the preprocessing pipeline, making the “Reusable” principle more concrete.

Furthermore, Sheehy et al. [68] emphasized the necessity of automating spectral analysis, focusing on the standardization of repetitive pre-processing procedures, which is a fundamental prerequisite for making Raman datasets truly “Reusable” and “Interoperable” according to the FAIR principles. They propose to test pipeline reproducibility by synthesizing new Raman spectra that combine a pure Raman signal (e.g., mathematically cleaned nylon spectrum), a known baseline (e.g., the spectrum of aluminum, a fluorescent material), and random noise (e.g., following a normal distribution). However, to date, the FAIR principles are still far from being achieved.

Closely connected to the standardization of computational pipelines is the rigorous design of the machine learning workflows themselves. The high dimensionality of Raman spectra, combined with the relatively small cohorts typical of early-stage feasibility studies [56,58,60], can lead to overoptimistic accuracy estimates if training and validation sets are not strictly separated [69]. The adoption of good validation strategies, together with confirmation in independent and multicenter cohorts, therefore represents a key opportunity to consolidate the reliability of the reported diagnostic accuracies and to support their transition from promising preliminary results to clinically validated tools [69].

A further point that warrants clarification is the distinction between conventional RS and SERS, which differ in their analytical principles and translational profiles. RS is intrinsically label-free and probes the global biochemical composition of the sample but suffers from an inherently weak signal that often requires long acquisition times [18]. SERS overcomes this limitation by exploiting plasmonic enhancement on metallic nanostructures, achieving sensitivity down to the attomolar range, but introduces additional sources of variability, including nanoparticle synthesis, substrate reproducibility, analyte orientation and surface chemistry, and batch-to-batch variability [41]. These differences are especially important in the context of clinical translation: label-free RS and antibody- or substrate-dependent SERS-LFA platforms should not be presented as sharing the same advantages and limitations, since the latter reintroduce a dependence on affinity reagents and on the reproducibility of the functionalized substrate [59].

Finally, additional steps toward routine clinical use include addressing inter-instrument and preanalytical variability [8,70] and benchmarking Raman-based metrics against established CSF and blood-based biomarkers through longitudinal, multicenter studies [71].

In line with this multiplex and system-level capability, Raman-based approaches have also been successfully applied to other complex diseases. In chronic obstructive pulmonary disease (COPD), SERS analysis of saliva has revealed disease-specific biochemical fingerprints associated with alterations in proteins, lipids, saccharides, and nucleic acids. In COPD, the combination of SERS with multivariate statistical analysis and machine learning enabled accurate discrimination between patients and healthy controls, as well as between different pathological conditions. Importantly, these spectral profiles were shown to correlate with clinical parameters, supporting the potential of Raman-based saliva analysis as a minimally invasive tool, not only for diagnosis but also for patient stratification and disease monitoring [72]. This translational potential was further demonstrated in oncology, where Raman-based analysis of serum and plasma samples enabled the discrimination between malignant and benign conditions with high diagnostic accuracy. Notably, longitudinal studies have shown that disease-associated spectral alterations can revert following tumor resection, directly linking Raman fingerprints to disease presence and highlighting the potential of RS not only for early diagnosis but also for therapy monitoring and follow-up [17].

Taken together, these features support the integration of Raman-based approaches into clinical workflows as minimally invasive, cost-effective tools for early diagnosis, patient stratification, and longitudinal monitoring of disease progression, particularly when combined with advanced data analysis pipelines.

In the framework of neurodegenerative disorders, among the approaches reviewed here, those already validated on clinical biofluids appear closest to clinical implementation within the next five years. In particular, label-free RS of saliva and serum represents the most realistic route toward a minimally invasive, low-cost screening tool [15,56], particularly suited to large-scale or first-line application. A systematic meta-analysis recently investigated the application of RS to AD diagnosis screening studies from multiple databases [52]. The analysis proved that RS achieves a pooled sensitivity of 0.86 positioning RS as a potentially non-invasive auxiliary diagnostic method for AD, with performance comparable to other and more conventional biomarker approaches [52].

## 7. Conclusions

Neurodegenerative diseases, such as PD and AD are being increasingly recognized as major public health concerns with profound implications for individuals, families, and societies worldwide. RS measures intrinsic molecular vibrations of a sample and can report on amyloid structure without the need for additional probes or pretreatment of the sample. The expanding clinical application of Raman techniques in the biomedical field has demonstrated its ability to identify pathological markers in accessible peripheral tissues like serum and saliva. The translation of RS to the neurodegenerative field is emerging and could lead to two major achievements: (1) to shed new light on the mechanisms of amyloid formation, trafficking, and neurotoxic spreading; (2) to develop Raman based platforms for the diagnosis and monitoring of neurodegeneration as well as for the monitoring of pharmacological and rehabilitation treatment. Although the theoretical basis has been laid, the full translation to routine clinical practice is still hampered by well-defined challenges related to the technological and clinical implementation. From a clinical point of view, the involvement of wide, independent, and multicenter cohorts, is mandatory to validate the Raman-based methodologies and to lead to concrete translatable solutions. On the other hand, standardizing sample handling procedures, harmonizing spectral preprocessing, and ensuring transparent reporting of machine learning workflows are essential for accumulating evidence and confirming the method’s substantial potential.

## Data Availability

No new data were created or analyzed in this study. Data sharing is not applicable to this article.

## Abbreviations

The following abbreviations are used in this manuscript:

Aβ	Amyloid-beta (amyloid-β)
AFM	Atomic Force Microscopy
AD	Alzheimer’s Disease
Ag	Silver (from Latin *Argentum*)
APP	Amyloid Precursor Protein
α-syn	Alpha-synuclein
Au	Gold (from Latin *Aurum*)
AUC	Area Under the Curve
BAC-SNCA	Bacterial Artificial Chromosome – SNCA transgenic
COPD	Chronic Obstructive Pulmonary Disease
CSF	Cerebrospinal Fluid
DLB	Dementia with Lewy bodies
FAIR	Findable, Accessible, Interoperable, Reusable
EVs	Extracellular Vesicles
ID	Intrinsically Disordered
LFA	Lateral Flow Assay
MCI-AD	Mild Cognitive Impairment due to Alzheimer’s Disease
NAC	Non-Amyloid-beta Component region
NIR	Near-Infrared Raman spectroscopy
NMR	Nuclear Magnetic Resonance
PD	Parkinson’s Disease
PFF	Preformed Fibrils
p-Tau181	Phosphorylated Tau at Threonine 181
RS	Raman Spectroscopy
SAA	Seed Amplification Assay
SERS	Surface-Enhanced Raman Spectroscopy
